# Adipose Tissue Uses in Peripheral Nerve Surgery

**DOI:** 10.3390/ijms23020644

**Published:** 2022-01-07

**Authors:** Allison Podsednik, Raysa Cabrejo, Joseph Rosen

**Affiliations:** 1 The University of Texas Rio Grande Valley School of Medicine, Edinburg, TX 78541, USA; allison.podsednik01@utrgv.edu; 2 Section of Plastic Surgery, Department of Surgery, Dartmouth-Hitchcock Medical Center, Lebanon, NH 03766, USA; raysa.g.cabrejo@hitchcock.org

**Keywords:** fat grafting, adipose-derived stem cell (ADSC), peripheral nerve surgery

## Abstract

Currently, many different techniques exist for the surgical repair of peripheral nerves. The degree of injury dictates the repair and, depending on the defect or injury of the peripheral nerve, plastic surgeons can perform nerve repairs, grafts, and transfers. All the previously listed techniques are routinely performed in human patients, but a novel addition to these peripheral nerve surgeries involves concomitant fat grafting to the repair site at the time of surgery. Fat grafting provides adipose-derived stem cells to the injury site. Though fat grafting is performed as an adjunct to some peripheral nerve surgeries, there is no clear evidence as to which procedures have improved outcomes resultant from concomitant fat grafting. This review explores the evidence presented in various animal studies regarding outcomes of fat grafting at the time of various types of peripheral nerve surgery.

## 1. Introduction

The concept of nerve surgery is astounding; we have developed the ability through microsurgery to suture the epineurium of one nerve, or of different nerves, together to promote the regeneration of the nerve and reinnervation of its structures. As early as 1873, surgeries to repair nerve gaps, such as the nerve flap, were being conceptualized [1]. However, World War I allowed for comparison of different nerve repair techniques on a larger scale, which led to more sound conclusions regarding nerve repair [1]. Though nerve surgery is now routinely performed, not all procedures have a superb success rate. Of people that undergo peripheral nerve surgery, only 40–50% are considered to have good functional recovery [2]. If a peripheral nerve incompletely heals, poor motor outcomes can occur, such as muscle denervation from the disruption of neuromuscular signaling, as well as poor sensory outcomes such as persistent pain syndromes. Regenerative medicine explores the use of novel biomaterials to enhance success rates and outcomes for patients undergoing nerve repair. One such biomaterial, adipose tissue, has been repurposed to enhance the healing of peripheral nerves following surgical repair. 

Following injury to a peripheral nerve, the distal stump will undergo Wallerian degeneration, while the proximal stump will exhibit central chromatolysis. The myelinating cells of the peripheral nervous system, Schwann cells, provide support to injured nerves attempting to regenerate via axonal sprouts and reinnervate distal structures. Adipose tissue contains adipose-derived stem cells (ADSCs), which can differentiate into many lineages [3]—but, most importantly to this paper, into Schwann-like cells—and assist in guiding the regeneration of axons [4,5]. ADSCs additionally supply regenerative potential to healing peripheral nerves by secreting various neurotrophic and angiogenic factors, including brain-derived neurotrophic factor (BDNF), glial-derived neurotrophic factor (GDNF), ciliary neurotrophic factor (CNTF), insulin-like growth factor 1, nerve growth factor (NGF), and neurotrophin (NT)-3 and -4 [6,7,8]. The paracrine effect of ADSCs on nerve regeneration has been evaluated in vivo and the factors secreted create a desirable microenvironment for the healing nerve. Enhancing the paracrine effect of ADSCs by controlling the release of neurotrophic factors such as BDNF or providing greater surface area for ADSCs has previously augmented ADSCs, and, on the contrary, the paracrine effect of ADSCs may be inhibited by materials introduced into the microenvironment that inhibit the dissemination of factors [9,10,11]. 

Schwann-like cells have been shown to migrate to sites of injury beyond the distal nerve segment, where they release nerve growth factors to promote nerve growth [7]. Molecules involved in Schwann cell morphological changes in response to nerve injury, such as c-Jun, allow for axoglial interactions and regeneration of axon tracts or bridges [12]. ADSCs can also secrete exosomes, which augment the production of myelin basic protein to increase the myelination of damaged peripheral nerves after demyelination has occurred [13,14]. The biology of adipose tissue, mostly due to the presence of ADSCs, disposes of adipose tissue to be used in surgical procedures to promote healing at the site of injury. 

Derivatives of adipose tissue include macrofat, microfat, nanofat, and microvascular fragments depending on the manipulation of the tissue. Macrofat and microfat are classically utilized as a filling biomaterial for soft tissue defects and injury, while nanofat is more often used in tissue remodeling because of its injectability and regenerative potential [15]. Conveniently, ADSCs can be applied as cell suspension injections or in combination with various biomaterials to assist in tissue regeneration [15]. Autogenous fat grafting is a method of delivering ADSCs without having to process the tissue, which avoids the FDA regulations that are placed on purified cells from adipose tissue [6]. In this manner, fat grafting is an accessible method that also keeps costs low, is minimally invasive, and does not pose the risk of an immune response [16]. 

## 2. Adipose Cells Enhance the Regeneration of Nerves 

### 2.1. Crush Injury Studies 

Crush injury studies are employed to reproduce nerve damage such as acute neuropathy. In these studies, a clamp is placed onto a nerve to provide pressure to, or “crush”, the nerve. The nerve experiences damage directly from the pressure and from the induced ischemia due to the comparatively smaller pressure of capillary perfusion relative to the externally applied pressure [17]. Though crushed, the nerve retains Schwann cells, which can assist in reinnervation efforts through the secretion of neurotropic factors. Neurapraxia or grade I nerve injury can result from acute or chronic compression. Neurapraxia is defined by focal demyelination in the absence of axonal or connective tissue damage and clinically presents as paresthesia and numbness [18]. Chronic nerve compression has worse outcomes compared to acute nerve compression at 4 weeks when both groups are treated with ADSCs [19].

A study on peroneal nerves in mice found that crush injuries treated with ADSCs in Matrigel exhibited greater functional recovery compared to injuries not treated with ADSCs as early as 2 days after surgery, as well as at 10 days [20]. In a crush injury study of 40 rats, white adipose tissue flap had positive effects both functionally and histologically on nerve regeneration. Groups treated with adipose tissue demonstrated significantly greater recovery maximum isometric tetanic force than untreated groups, indicating better functional recovery [21]. Adipose-tissue-treated nerves also had a 13% increase in axon number (*p* < 0.05) and 31% increase in myelin thickness/axon width ratio (*p* < 0.001), indicating better histological recovery [21]. Another study injected ADSCs distal to the site of rat sciatic nerve 14-day crush injuries, which resulted in less muscle atrophy as well as better functional and anatomical outcomes than the group that had the compression clip relieved after 14 days with no subsequent epineural ADSC injection [17]. Animal experiment results presented in the literature support the continued exploration of ADSC addition to crush nerve injuries.

### 2.2. Quality of Regeneration 

Regeneration of the nerve is important, but it is also important to limit scarring during the healing process. Perineural adherences can derail proper healing after peripheral nerve surgery and result in persistent pain. ADSCs can prevent damage to the nerve while facilitating nerve regeneration. In a study of sciatic nerve surgical injury in mice, fat grafting reduced scar tissue development around the healing nerves [22]. Thus, adipose tissue not only can mechanically protect nerves during the healing process by acting as a barrier but can act as a biological compound with pro-regenerative properties [22]. Recently, ADSCs have been shown to constitutively produce exosomes that contain neural growth factor transcripts [23]. The exosomes released by ADSCs create desirable microenvironmental conditions to contribute to neurite regeneration via axon outgrowth. Schwann cells from nerve injury sites cocultured in vitro with ADSCs with varying levels of ADSC-derived exosomes demonstrated that increased exosome levels correlated with decreased apoptosis and increased proliferation of Schwann cells [24]. The Schwann cells that experienced decreased apoptosis expressed relatively more Bcl-2 mRNA and less Bax mRNA. Preventing scar tissue formation, which decreases peri-neural adherences, is clinically important, especially when considering hand surgery. Carpal tunnel release and Dupuytren’s contracture surgery both rely on the unstressed gliding of the nerves and tendons for surgical success. Preventing adherences would facilitate greater surgical success and allow for more smooth hand movements. In a study evaluating the capability of muscarinic receptors on modulating the nerve growth factor production of rat ADSCs, M2 receptor stimulation reduced apoptotic factor proNGF-B, which demonstrates that acetylcholine promotes the maturation of NGF [25]. There are likely other molecules in addition to acetylcholine that can be targeted to improve the regenerative properties of ADSCs. ADSC-derived exosomes have experimentally been manipulated to carry more NT-3 mRNA, which improved nerve recovery in rat models after two weeks [26]. Hypoxic expansion has also been shown to increase the neuronal differentiation potential of ADSCs in vitro [27]. Additionally, theorized but not yet clinically evaluated, β_2_ receptor stimulation of peripheral nerves leads to an increase in cAMP, which is a molecule that has been associated with Schwann cell proliferation [28,29]. Aiding Schwann cell function is another approach that has potential to lead to better-quality nerve regeneration outcomes.

## 3. Animal Studies Utilizing Fat in Various Types of Peripheral Surgery 

### 3.1. Nerve Repair 

Nerve primary tensionless repair is indicated after acute transection of a nerve. For nerve transections that can be repaired without undue tension, the gold standard is direct epineural coaptation with micro sutures, though tissue adhesives may be used in place of or in adjunct to micro sutures [30]. To avoid unwanted tension, end-to-end neurorrhaphy should only be used in humans if the nerve gap is 1 cm or less [31]. Following injury to a peripheral nerve, Schwann cells are activated to enter a repair program state to perform functions including the activation of negative regulators of myelination to demyelinate the damaged nerve, modulation of genes to promote neuron survival and axonal regrowth, and remyelination of the regenerated axon [12]. Though Schwann cells naturally act to promote peripheral nerve regeneration and healing, natural repair without surgical manipulation has numerous obstacles. Schwann cells modulate genes only transiently, so, as time passes, the Schwann cells provide less assistance to the nerve, whether it healed properly or not [32]. Regenerating axons are guided to their targets, but sometimes fail to enter the correct endoneurial tube and do not successfully reinnervate the target organ. Surgical coaptation of the transected nerve ends can help to prevent errors in reinnervation, but still requires guidance from the Schwann cells to heal the nerve after coaptation. Even then, only around 10% of axons reach the intended target muscle or organ after surgical repair following transection [33]. Nerve transections are associated with poor outcomes even after repair, especially when the distance between the nerve and target is long or when repair is performed after a delayed period [34]. Consequently, only transections that can be repaired without undue tension should be attempted without a graft and repairs should take place as soon as possible. Providing extra materials to guide the regenerating axons, such as ADSCs present in adipose tissue that can transform to Schwann-like cells, could be of benefit to nerves repaired by direct coaptation with suture. 

A study of rat sciatic nerve injury managed to map cell ADSC migration in vivo. After nerve injury, approximation of the stumps with nerve suture was combined with ADSC injection, where ADSCs were tracked for 14 days. The experiment confirmed that ADSCs migrated in vivo to the distal section of the nerve injury from the proximal injection site within two weeks, likely guided by chemotactic factors, which coincided with observed nerve regeneration and functional recovery [35]. Not only has ADSC migration been concurrent with regeneration but primary nerve repair in rats demonstrated better regeneration with fat grafting compared to primary nerve repair alone according to sciatic function index and pin prick results [36]. The injected fat was centrifuged autologous fat that received no chemical manipulation, which demonstrates the raw regenerative potential of adipose tissue. A slightly different introduction of ADSCs showed similarly promising results; when ADSCs were suspended in fibrin glue, the addition to epineural suture repair after transection in rat sciatic nerves of the enhanced glue was significantly superior in terms of myelinization, axon count, and muscle weight quotient than that of normal fibrin glue [37]. Using fibrin glue has the additional benefit of providing extracellular support to the healing nerve. Another study evaluated the functional recovery of rat sciatic nerves after repair with and without ADSCs. A swim test revealed accelerated functional recovery at 2 weeks, with continued improvement at 6 weeks, for ADSC-treated repairs compared to non-treated repairs [33]. Upon histological analysis, the ADSC-injected group had significantly higher mean values for nerve fiber density, axon area, and myelin area [33].

Of the studies discussed, only Schweizer, Dong, and Tuncel evaluated functional outcome measures after nerve repair with ADSC addition. While the other studies provide convincing histological evidence of improvement, it is important to demonstrate that histology translates to a functional benefit. In this way, demonstrating a functional outcome benefit provides a higher level of evidence. Of the papers that evaluated functional outcomes in Table 1, Schweizer’s employment of the swim test alongside other commonly tested functional indices provides strong support of the reported outcomes, while Tuncel’s study sample size ensures the accuracy of the reported results. 

### 3.2. Nerve Grafting

The peripheral nervous system (PNS) possesses regenerative ability due to the presence of Schwann cells versus oligodendrocytes in the central nervous system and the continuous basement membrane on the outer surface of Schwann cells, termed the neurilemma [38]. However, in some more severe cases of injury, the PNS is unable to regenerate axons. The longer a nerve must travel to be fully regenerated, the slower the rate of regeneration. This phenomenon has to do with the distance from the cell body, which is where the cell machinery working to make new proteins is located, and then the cell will have to transfer these materials down its axons. There is a specific gap length, the critical sized nerve gap, for each species in which the space is too large for a peripheral to be able to regenerate or reinnervate targets. Nerve gaps under the threshold number can successfully be repaired using a nerve graft [7]. The rat critical nerve gap length is 1.5 cm and human is 4 cm. A human PN will naturally regenerate 1 mm/day, and animal nerves are assumed to regenerate at this rate as well. Autologous nerve grafts are the most reliable method of reconstructing nerve gaps, especially for sensory nerve gaps greater than 3 cm or for any motor nerve [30]. Acellular grafts alone have not been as effective as autografts for longer grafts and the gold standard for motor nerves is to use a sensory nerve as a graft [39]. The clinical repair rate neared 80% for nerve injuries repaired with autologous nerve grafts over a decade ago [40]. Not all nerves repaired with grafts should be expected to have the same recovery rate. With intermediate-level repair in relation to the proximal stump, motor recovery potential was significantly greater for musculocutaneous (100%), radial (98.3%), and femoral (87.5%) nerves compared to tibial (63.9%), median (52%), and ulnar (43.6%) nerves [41]. In this way, the authors speculated that the characteristics of the effector muscle, nerve microanatomy, and topography of motor neurons within the spinal cord are the differentiating factors [41]. 

In a study where nerve grafts taken from the other leg of the same rat were used to repair sciatic nerve injuries, ADSC transplant demonstrated improved regeneration. Specifically, the ADSCs increased the survival of spinal L5 ganglia neurons by 26.4%, improved sciatic nerve vascularization by 35.68% and increased the number of myelin fibers in the distal nerve by 41.87% [42]. In a separate study, after transection and autologous graft repair in rats, an experimental group that received fibrin glue containing ADSCs after sutures had an 18% higher amount of myelin fibers in the distal nerve segment than the group that received fibrin glue without ADSCs 30 days after the surgery [43]. Finally, in an experiment involving facial nerve lesion in rats, the sciatic nerve was used as a graft to connect the left and right marginal mandibular branches of both facial nerves. A group that was wrapped in an ADSC sheet had significantly higher amplitude when evaluated with evoked compound electromyography compared to both the group that received an ADSC suspension after the graft and the group that served as the control, with only a graft and no ADSC supplementation [44]. The ADSC sheet group also had a significantly reduced the time needed for reinnervation compared to the other two groups [44]. As seen in Table 2, both Masgutov and Fujii correlated histological outcomes with functional outcomes, which provides powerful evidence of the benefit provided by ADSCs in nerve grafting surgeries. 

#### TENGs

Repair of longer nerve gap lengths that extend beyond the critical sized nerve gap (4 cm in humans) proves difficult. One strategy to improve success for long nerve gap length repairs involves the addition of enhancing agents such as Schwann cells, stem cells, and growth factors [45]. Over the last couple of decades, various types of scaffolds, such as acellular nerve grafts (ANGs), nerve guidance conduits, and non-nervous tissues, have been filled with Schwann cells, stem cells, and/or neurotrophic factors to develop tissue-engineered nerve grafts (TENGs) [46]. Bioactive materials such as silk fibers have been explored as nerve conduits [47] in conjunction with ADSCs and demonstrated promotion of nerve regeneration [48]. Though acellular grafts alone have proven less efficacious than autografts with longer graft distances, enhanced acellular grafts and autografts are comparable [39,49,50]. Especially for longer distances, TENGs can be an alternative to autografts and have the added benefit of no donor nerve sacrifice for graft donation purposes and avoidance of nerve mismatch [51]. As discussed previously, grafts are necessary once injury-caused gaps are larger than the critical sized gap but can be used on smaller gaps as well to avoid creating tension. Nerves regenerate better when an intact endoneurium is present [18,52], though a nerve guidance conduit alone has not been successful in a gap larger than 1.3 cm in rats, which is a non-critical gap length [7]. Bridging larger lengths requires more than the mechanical guidance of nerve ends being enclosed in a tube—the tube needs to provide biological assistance to the regenerating axons. TENGs, being lined with biological or synthetic materials, provide the necessary regenerative assistance to the nerve. 

In a study on rat 8 mm facial nerve gaps, the outcomes of autografts, decellularized artery conduit, and decellularized artery seeded with ADSCs were compared [53]. The conduits seeded with ADSCs had significantly superior restoration outcomes compared to the conduits alone; however, the autographs had recovery of whisking, maturation of myelinated fibers, and number of facial neurons that was significantly greater than either of the two conduit groups [53], supporting the idea that autographs are the gold standard at non-critical length gaps. A study in which 10 mm rat sciatic nerve defects were bridged with a xenogeneic acellular nerve matrix seeded with differentiated ADSCs showed that regeneration occurred by electrophysiological and histological standards [54]. In studying more closely the cellular mechanisms, the ADSCs were observed to align on the longitudinal axis of the matrix, which had preserved basal lamina and a porous microstructure, to resemble bands of Bünger, which guide regrowing axons and express NGF, GDNF, and BDNF [54]. Another experiment using rats compared the outcomes of multiple conditions including an autograft, a silicone conduit seeded with ADSCs, and a silicone conduit seeded with differentiated ADSCs to repair 10 mm sciatic nerve gaps. After 6 months, both the groups that received ADSCs and differentiated ADSC-seeded conduits displayed significant improvements according to the sciatic function index, as well as higher mid and distal nerve myelin fiber density compared to the other groups in the study [55]. A comparable experiment showed that, in 7 mm rat facial nerve defects autografts, silicone conduits with undifferentiated ADSCs, silicone conduits with differentiated ADSCs, and silicone conduits with Schwann cells all had similar efficacies [56]. Interestingly, stromal vascular fraction (SVF) could have benefit versus ADSCs in facial nerve defects, though both SVF and ADSCs resulted in efficacious nerve regeneration in a rat study [57]. An additional experiment showed that type I collagen conduits seeded with undifferentiated ADSCs used to treat 10 mm rat sciatic nerve gaps had higher motor and sensory nerve conduction velocity values than conduits alone after 6 months [58]. The studies that provide functional evaluations in this set include Sun, Orbay, and Watanabe (see Table 3). While Sun’s experiment maintained that autografts are the gold standard for smaller nerve gaps, Orbay and Watanabe demonstrated a benefit in functional outcomes when TENGS filled with ADSCs were used to repair small nerve gaps. Overall, this set of experiments supports the idea that TENGs are beneficial even at non-critical lengths, though more studies evaluating functional outcomes with increased numbers of subjects are needed. 

Fewer experiments have been performed evaluating ADSC-enhanced conduits at critical nerve lengths. In a rat study, empty nerve conduits and nerve conduits seeded with differentiated ADSCs were compared as treatment methods for 15 mm sciatic nerve gap defects [59]. The stem-cell-enhanced conduits demonstrated a 3.5-fold greater proportion of axons in the distal nerve stump than the empty conduits after 8 weeks [59]. More studies on long nerve defects are needed, especially experiments that evaluate functional outcomes and their correlation with histological results. One experiment demonstrated impressive results, though MSCs instead of ADSCs were used. Acellular nerve conduits seeded with MSCs demonstrated structural and functional repair of a 40 mm ulnar nerve gap defect in rhesus monkeys after 6 months [60]. Hu’s experiment provides relatively stronger evidence than rat experiments due to their use of non-human primates, which have genomes more similar to humans, therefore providing more applicable results [61]. Though more animal experiments need to be done to demonstrate the functional benefit, the initial experiments using stem cells, including ADSCs, to enhance nerve conduits in critical length nerve defects show promising results. 

**Table 3:** Participant number and outcome evaluations from studies of animal nerve surgeries with use of TENGs and ADSCs. Note that Hu’s study used MSCs instead of ADSCs. Only studies that evaluated at least one functional outcome are listed in the table.

Surgical Repair Method	Study	Participants (n)	Evaluations
TENGs	Sun [53]	10	Compound muscle action potentials, myelinated nerve fiber count, muscle weight, muscle fiber diameter measurement
	Orbay [55]	5	Walking track analysis, nerve conduction velocity, myelinated nerve fiber and vascular count, in vivo immunofluorescence
	Watanabe [56]	16	Myelinated axon fiber count, modified facial palsy scoring system to evaluate functional recovery
	Hu [60]	6	Compound muscle action potential, nerve conduction velocity, neurofilament positive axon count

### 3.3. Nerve Transfer

Nerve transfers should be considered for proximal nerve injuries and in injuries where there is concern that degradation of neuromuscular junctions will occur before reinnervation is possible [30]. Transfers are classically used in brachial plexus injuries. Proximal injury to a nerve necessitates regenerating axons from the proximal stump to travel farther, which makes reinnervation of the distal target more difficult. In fact, in a paper that assessed the variability of nerve graft success, the proximal grafts were highly unsuccessful compared to intermediate and distal grafts [41]. Performing a nerve transfer provides regenerating axons to the distal end of the injured nerve, bypassing the problem as well as overcoming graft length limitations [62]. 

ADSCs have been experimented with in animal studies involving nerve transfers. In a study of rats, a cross-facial nerve graft, which is classified as a transfer, was performed by coapting a transected facial nerve to both ipsilateral and contralateral buccal branches, using the sural nerve as an interpositional graft to bridge the distance [63]. In one group, ADSCs were injected after the transfer was performed, and another group did not receive ADSC injection. The ADSC-injected group showed enhanced axonal regeneration evidenced by a statistically significantly increased improvement in whisking behavior compared to the control group [63]. In an animal experiment in rats involving TENGs, brachial plexus injuries were repaired using a contralateral C7 nerve transfer combined with either unenhanced acellular nerve allografts or with acellular nerve allografts seeded with differentiated ADSCs [64]. The group that received differentiated ADSCs showed improved compound muscle action potential and motor conduction velocity as well as histologically more neurofilament, S100, larger diameter axons, thickened myelin sheaths, and higher-density myelination [64]. Another study transferred the phrenic nerve to restore the musculocutaneous nerve in C5-C6 avulsion injury in rats but instead used bone marrow stem cells in the treatment group to seed acellular nerve grafts [65]. Interestingly, there were no differences reported between groups with acellular nerve grafts and groups where acellular nerve grafts were seeded with bone marrow stem cells (BMSCs). Previously, both BMSCs and ADSCs have demonstrated the ability to differentiate into Schwann-like cells [4], so it is unclear why ADSCs showed histological and functional advantages in nerve transfer experiments when BMSCs did not. However, even in the case that BMSCs and ADSCs were to demonstrate similar outcomes in experiments, ADSCs have the benefit of being more easily accessible. As seen in Table 4, the studies by Abbas and Yang both evaluated functional outcomes and correlated them to histological observations, which builds a strong level of evidence. Additionally, it should be noted that ADSCs demonstrated functional benefits in nerve recovery in both the face as well as the brachial plexus. More experiments that evaluate functional nerve recovery should be performed in both facial nerves and the brachial plexus to corroborate the results.

## 4. Conclusions

Animal experiments demonstrate that various types of peripheral nerve surgeries have the potential to benefit from the addition of ADSCs during surgery. ADSCs have proven beneficial for nerve regeneration on multiple levels, including by secreting growth factors and by morphing into Schwann-like cells, which can modulate genes in a way that facilitates peripheral nerve healing. Though the experiments that have been performed are encouraging, more experiments are needed to demonstrate the undeniable benefit and in which settings they are beneficial. Very few of the existing studies evaluate the functional outcomes of ADSC addition to peripheral nerve studies, as seen in this review. One of the reasons that animal studies are so useful is due to their ability to correlate histological and functional outcomes, which is not ethical in many cases in human studies. Future studies should consider evaluating functional outcomes so that meaningful applications could be more easily extracted in relation to physiological effects. Experiments involving human peripheral nerves do exist because ADSCs are relatively safe, though most are small case studies. There is a need for randomized control trials evaluating the outcomes of peripheral nerve surgery with the addition of ADSCs. 

## Figures and Tables

**Table 1:** Participant number and outcome evaluations studies of animal nerve repair surgeries with use of ADSCs. Only studies that evaluated at least one functional outcome are listed in the table.

Surgical Repair Method	Study	Participants (n)	Evaluations
Nerve Repair	Schweizer [33]	10	Swim test, static sciatic index, toe spread factor
	Dong [35]	4	EMG, mechanical pain threshold
	Tuncel [36]	72	Walking track analysis, sciatic functional index, pin prick

**Table 2:** Participant number and outcome evaluations from studies of animal nerve grafting surgery with use of ADSCs. Only studies that evaluated at least one functional outcome are listed in the table.

Surgical Repair Method	Study	Participants (n)	Evaluations
Nerve Grafting	Masgutov [43]	5	Sciatic functional index, compound muscle action potentials, laser Doppler, fluorescence studies
	Fujii [44]	8	Facial Palsy Scoring System, EMG, myelinated nerve fiber count

**Table 4:** Participant number and outcome evaluations from studies of animal nerve transfers with use of ADSCs. Only studies that evaluated at least one functional outcome are listed in the table.

Surgical Repair Method	Study	Participants (n)	Evaluations
Nerve Transfer	Abbas [63]	6	Vibrissae motor performance, EMG, myelinated axon fiber count, immunohistochemical analysis of neuromuscular junction
	Yang [64]	10	Modified grooming test to evaluate shoulder rotation and abduction as well as elbow flexion, compound muscle action potentials, myelinated axon fiber count, histological analysis
